# Elevation of MMP-9 and IDO induced by pancreatic cancer cells mediates natural killer cell dysfunction

**DOI:** 10.1186/1471-2407-14-738

**Published:** 2014-10-02

**Authors:** Yun-Peng Peng, Jing-Jing Zhang, Wen-biao Liang, Min Tu, Zi-Peng Lu, Ji-Shu Wei, Kui-Rong Jiang, Wen-Tao Gao, Jun-Li Wu, Ze-Kuan Xu, Yi Miao, Yi Zhu

**Affiliations:** Department of General Surgery, The first Affiliated Hospital of Nanjing Medical University, 300 Guangzhou Road, Nanjing, 210029 People’s Republic of China; Jiangsu Province Academy of Clinical Medicine, Institute of Tumor Biology, 300 Guangzhou Road, Nanjing, 210029 People’s Republic of China; Transfusion Laboratory, Jiangsu Province Blood Center, 183 Longpan Road, Nanjing, 210042 People’s Republic of China

**Keywords:** Pancreatic cancer, Natural killer cell dysfunction, MMP-9, IDO

## Abstract

**Background:**

Natural killer (NK) cells play a key role in non-specific immune response in different cancers, including pancreatic cancer. However the anti-tumor effect of NK cells decreases during pancreatic cancer progression. The regulatory pathways by which NK cells facilitate tumor immune escape are unclear, therefore our purpose was to investigate the roles of the contributory factors.

**Methods:**

NK cells isolated from fresh healthy peripheral blood were co-cultured with normal human pancreatic ductal cells hTERT-HPNE and human pancreatic cancer cell lines SW1990 and BxPc-3 *in vitro*. Then NK cell function was determined by Flow cytometric analysis of surface receptors and cytotoxic granules in NK cells, NK cell apoptosis and cytotoxicity, and Enzyme-linked immunosorbent assay of cytokines. Expression level of MMP-9, IDO and COX-2 in hTERT-HPNE and SW1990 cells were detected by quantitative RT-PCR. Statistical differences between data groups were determined by independent *t*-tests using SPSS 19.0 software.

**Results:**

Our results showed that NK cell function was significantly downregulated following exposure to pancreatic cancer cells compared to normal pancreatic cells, as demonstrated by lower expressions of activating surface receptors (NKG2D, DNAM-1, NKp30 and NKp46) and cytotoxic granules (Perforin and Granzyme B); decreased secretion of cytokines (TNF-α and IFN-γ); and reduced cytotoxicity against myelogenous leukemia K562 cells. Further investigations revealed that MMP-9 and IDO may be implicated in SW1990 cell-induced NK cell dysfunction by facilitating tumor immune evasion. Blockade by TIMP-1 and/or 1-MT could partially restore NK function.

**Conclusions:**

Taken together, elevation of MMP-9 and IDO induced by pancreatic cancer cells mediates NK cell dysfunction. Our findings could contribute to the development of NK cell-based immunotherapy in patients with pancreatic cancer.

## Background

Pancreatic cancer is one of the most common gastrointestinal malignancies worldwide and is the fifth leading cause of cancer-related deaths [1]. It is characterized by rapid progression and intrinsic and acquired drug resistance [2]. Pancreatic cancer is difficult to diagnose in its early stages, therefore the rate of surgical resection is low (<20%) [3] and the five-year survival rate is poor (<6%) [1]. The progression of pancreatic cancer is affected by the surrounding microenvironment which largely consists of cancer associated fibroblasts and infiltrating inflammatory cells [4–6], including natural killer (NK) cells.

NK cells are key components in the innate immune system, acting as the first line of defense in the body. They are a type of cytotoxic lymphocyte that mediate a pro-inflammatory response and are closely associated with the progression of cancer, including pancreatic cancer [7]. Evidence shows that NK cells not only kill target cancer cells directly, without prior sensitization [8, 9], but also by binding to specific surface ligands expressed on the surface of cancer cells, such as major histocompatibility complex class I (MHC class I) molecules [10]. The effectiveness of NK cells as anticancer agents depends not only on the number of circulating, activated NK cells [11] but also on the expression of activating surface receptors, inhibitory surface receptors, cytotoxic granules and cytokines. NK cell dysfunction has been reported in several cancers, such as pancreatic cancer [7], gastric cancer [12], and colorectal cancer [13]. Although cancer cells have been shown to induce NK cell dysfunction *via* downregulation of specific activating surface receptors (e.g. NKG2D), natural cytotoxicity receptors (NCR) [14], cytotoxic granules (e.g. Perforin and Granzyme B) [15], decreased secretion of cytokines (e.g. TNF-α and IFN-γ) [16] and upregulation of inhibitory surface receptors (e.g. KIR3DL1 and KIR2DL1/DS1) [17, 18], the underlying mechanisms are largely unknown.

Several studies have suggested that matrix metallopeptidase 9 (MMP-9), a 92-kDa type IV collagenase which is secreted by mesenchymal stem cells (MSC), can significantly downregulate the cytotoxicity of NK cells *in vitro* by targeting T cells [19, 20]. Indoleamine 2, 3-dioxygenase (IDO) has also been reported to play a role in MSC-mediated immunosuppression [21] by inhibiting NK cell accumulation and suppressing NK cell function [22]. This suggests that MMP-9 and IDO may play similar roles in tumor-induced NK cell dysfunction. Decreased infiltration of inflammatory cells into the tumor microenvironment has been associated with increased expression of COX-2 [23], which is known to promote tumor growth *via* its major product prostaglandin E2 (PGE2) in a T cell or NK cell-dependent manner [24, 25]. Together, MMP-9, IDO and PGE2 are potent effectors in the interaction between pancreatic cancer cells and NK cells. The mechanism by which they promote NK cell dysfunction is the focus of this investigation.

## Methods

### Antibodies and reagents

Anti-human CD3-FITC/CD16 + 56-PE mixed antibody were purchased from Beckman Coulter (Brea, CA, USA). Anti-human CD16-FITC, NKG2D-PE, NKp44-APC, DNAM-1-FITC, NKp46-Alexa Fluor 647, NKp30-PE, KIR3DL1-FITC, KIR2DL1/DS1-PE, NKp30-APC, Perforin-PE and Granzyme B-FITC antibodis were all purchased from BioLegend (San Diego, CA, USA), as well as Fixation Buffer, Wash Buffer, Annexin V binding buffer, Alexa Fluor 647 Annexin V and 7-AAD Viability Staining Solution. The MPP-9 and IDO inhibitors were 1-Methyl-DL-tryptopan (1-MT; Sigma-Aldrich, St. Louis, MO, USA) and Tissue inhibitor of metalloproteinases 1 (TIMP-1; PeproTech, Rocky Hill, NJ, USA). Human NK Cell Isolation Kit was purchased from Miltenyi Biotec (Auburn, CA, USA) and ELISA kits were purchased from Abcam (Cambridge, MA, USA). Trizol reagent and PrimeScript RT Master Mix (Perfect Real Time) were both obtained from TaKaRa (Shiga, Japan) and Power SYBR Green PCR Master mix was purchased from Applied Biosystems (Carlsbad, CA, USA).

### NK cell isolation

Fresh peripheral blood samples from healthy volunteers were provided by Jiangsu Province Blood Center (Gu Jian, China). Peripheral blood mononuclear cells (PBMC) were isolated by Ficoll-Hypaque density gradient centrifugation. NK cells were selected from the PBMCs by negative magnetic selection. The purity of the NK cells was >92%, as quantified by multicolor flow cytometry (Gallios; Beckman Coulter).

### Cells and cell culture

The normal human pancreatic ductal cell line hTERT-HPNE and pancreatic cancer cell lines SW1990 and BxPc-3 were obtained from the American Type Culture Collection (ATCC; Rockville, MD, USA). The hTERT-HPNE cells were cultured as previously described [26]; SW1990 and BxPc-3 cell lines were cultured in DMEM supplemented with 10% FBS, penicillin (100 U/mL) and streptomycin (100 μg/mL). The myelogenous leukemia K562 cell line (ATCC) was cultured in RPMI 1640 supplemented with 10% FBS, penicillin (100 U/mL) and streptomycin (100 μg/mL). The NK-92 cell line was kindly donated by Professor Bin Gao and was cultured as previously described [27]. Purified NK cells were cultured in 6-well plates (3 × 10^5^ cells/well) in AIM-V media supplemented with 10% FBS, penicillin (100 U/mL), streptomycin (100 μg/mL) and interleukin 2 (IL-2; 100 U/mL; PeproTech, Rocky Hill, NJ, USA), either in absence or presence of hTERT-HPNE, SW1990 and BxPc-3 cells (5 × 10^5^ cells/well). In order to investigate the roles of MMP-9 and IDO, NK-92 cells were co-cultured with SW1990 cells in 6-well plates (NK-92/SW1990 ratio: 3 × 10^5^/5 × 10^5^ cells/well) in the presence of 0.5 ug/ml TIMP-1 (a specific blocker for MMP-9) and/or 0.5 mM 1-MT (a specific blocker for IDO).

### Flow cytometric analysis

NK and NK-92 cells, either cultured alone or co-cultured with normal or cancer pancreatic cell lines, were harvested after five days and divided into four tubes, labeled as T1, T2, T3 and T4. Firstly, T1, T2, T3 and T4 was respectively washed twice with PBS. Secondly, T1 was stained with anti-human CD16-FITC, NKG2D-PE, and NKp44-APC antibodies; T2 was stained with anti-human DNAM-1-FITC, NKp30-PE, and NKp46- Alexa Fluor 647 antibodies; T3 was stained with anti-human KIR3DL1-FITC, KIR2DL1/DS1-PE and NKp80-APC antibodies; T4 was added with 500 μl fixation Buffer and incubated in the dark at room temperature for 20 min, and then, T4 was washed twice with Wash Buffer and stained with anti-human Granzyme-B-FITC, and Perforin-PE antibodies. After incubating in the dark at room temperature for 15 min, the cells were washed twice with PBS. After staining, the tubes were incubated in the dark at room temperature for 15 min and washed twice with PBS. Lastly, all tubes were analyzed by multicolor flow cytometry. Data were analyzed using Kaluza software.

### Apoptosis of NK cells

NK cells, either cultured alone or co-cultured with normal or cancer pancreatic cell lines, were harvested after three days and resuspended in 500 μL Annexin V binding buffer. The cells were stained with 5 μL Alexa Fluor 647 Annexin V and 7-AAD Viability Staining Solution and incubated in the dark at room temperature for 15 min before being analyzed by multicolor flow cytometry.

### Apoptosis of K562 cells

NK and NK-92 cells, either cultured alone or co-cultured with normal or cancer pancreatic cell lines, were harvested after three days. K562 cells were treated with the harvested NK or NK-92 cells were in the presence of IL-2 (100 U/mL) for 2 h at different effector-to-target (E/T; NK/K562) ratios (1:1, 3:1, 9:1). The cells were then collected and stained with CD3-FITC/CD(16 + 56)-PE, incubated in the dark at room temperature for 15 min, washed 2 times with PBS and resuspended in 500 μL Annexin V binding buffer. The cells were stained with 5 μL Alexa Fluor 647 Annexin V and 7-AAD Viability Staining Solution and incubated in the dark at room temperature for a further 15 min. According to cells staining, cell subset which detected as CD3-/CD(16 + 56) + was NK cells, and another cell subset which detected as CD3-/CD(16 + 56)- was K562 cells. Percentage of apoptosis K562 cells before was analyzed by multicolor flow cytometry.

### Enzyme-linked immunosorbent assay (ELISA)

The expression of some protein (IDO, MMP-9, GM-CSF, TNF-α, and IFN-γ) were detected by ELISA. The concentrations of MMP-9 and IDO in cell culture supernatants (NK cells cultured alone, hTERT-HPNE cells cultured alone or co-cultured with NK cells, SW1990 cells cultured aloneor co-cultured with NK cells; and NK-92 cells cultured alone or co-cultured with SW1990 cells in the absence or presence of TIMP-1 and/or 1-MT) were determined by specific ELISA kits. The concentrations of GM-CSF, TNF-α and IFN-γ in cell culture supernatants (NK cells cultured alone or co-cultured with hTERT-HPNE and SW1990 cells; and NK-92 cultured alone or co-cultured with SW1990 cells in the absence or presence of TIMP-1 and/or 1-MT) were also determined. All procedures were carried out according to the manufacturer’s protocols.

### Quantitative real-time reverse-transcription polymerase chain reaction (qRT-PCR)

Total RNA was extracted from hTERT-HPNE or SW1990 cells cultured alone or co-cultured with NK cells using Trizol reagent and reverse-transcribed to cDNA using PrimeScript RT Master Mix (Perfect Real Time). The cDNA was amplified using Power SYBR Green PCR Master mix and the Step One Plus Real-Time PCR System (Applied Biosystems, Carlsbad, CA, USA). Each procedure was performed according to the manufacturer’s instructions. The sequences of gene specific primers for human MMP-9, IDO, COX-2, and β-actin were designed and purchased from Invitrogen (Carlsbad, CA, USA) using Primer Premier 5 and checked by Oligo 6 (Invitrogen). Expression levels were normalized relative to the cell line according to the formula 2^-ΔΔCt^, where Ct is the cycle threshold.

### Statistical analysis

Statistical differences between data groups were determined by independent *t*-tests using SPSS 19.0 software (SPSS Inc., Chicago, IL, USA). Data are expressed as mean ± SD; *P* < 0.05 was considered statistically significant.

## Results

### Pancreatic cancer cells altered the percentages of surface receptors and cytotoxic granules in NK cells

**Figure 1:**
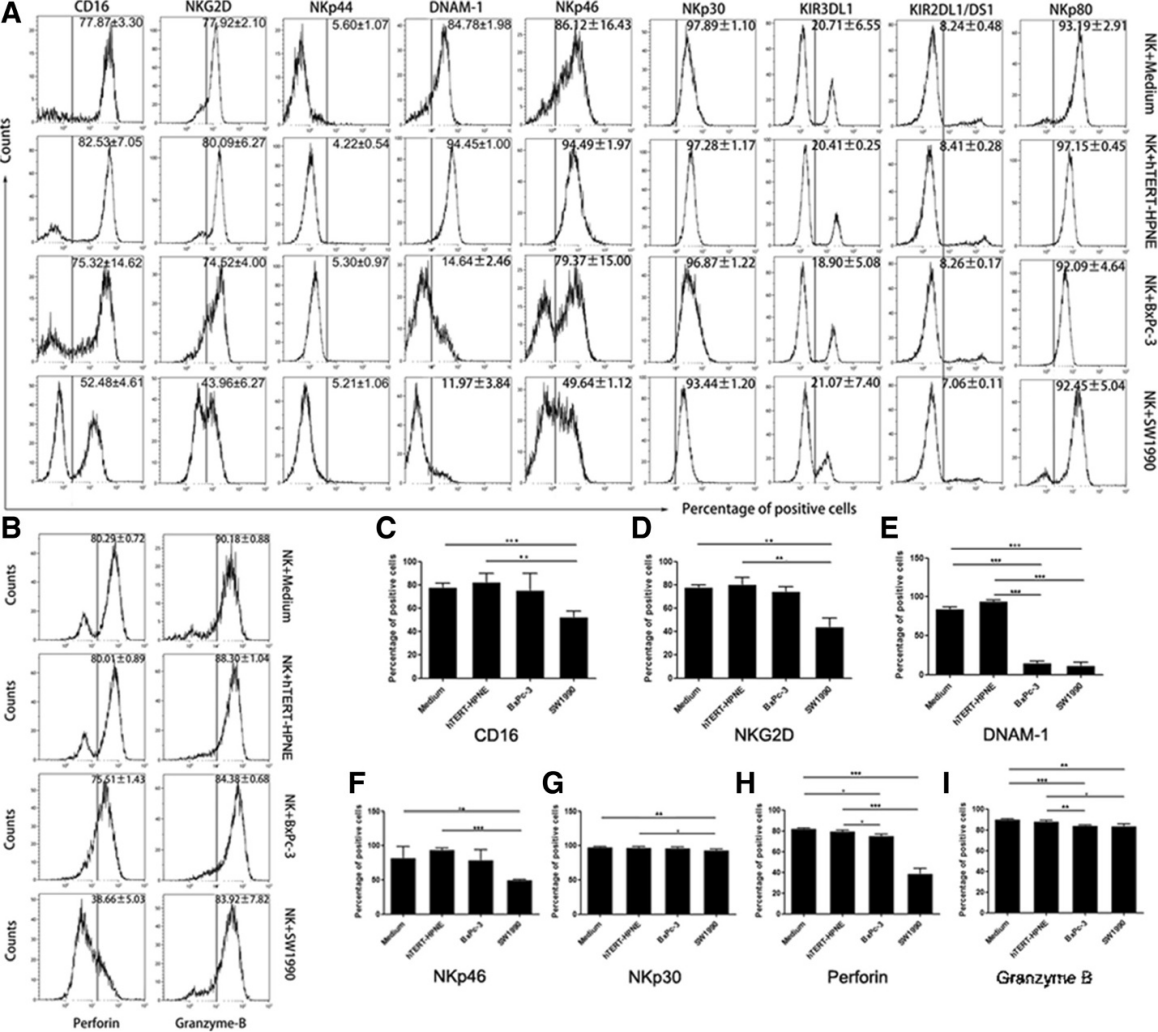
**Comparisons between NK cells cultured alone and those cultured with normal pancreatic hTERT-HPNE cells or pancreatic cancer BxPc-3 and SW1990 cells. (A and B)** The percentages of surface receptor-positive and cytotoxic granule-positive NK cells harvested from the different NK cell-pancreatic cell cultures. **(C–** **I)** Comparisons between the percentages of positive NK cells in the different culture groups. All experiments were repeated three times. All data were expressed as mean ± SD. Independent t-tests were used for statistical analysis through SPSS 19.0 software. *represents P <0.05, **represents P <0.01, and ***represents P <0.001.

Nine surface receptors (CD16, NKG2D, DNAM-1, NKp30, NKp44, NKp46, NKp80, KIR3DL1, and KIR2DL1/DS1) and two cytotoxic granules (Perforin and Granzyme-B) had been selected for investigation. Their levels and the percentages of NK cells positive for each of these proteins were determined after the NK cells had been cultured alone or with hTERT-HPNE, BxPc-3 or SW1990 cells (Figure 1).

The percentages of NK cells positive for CD16, NKG2D, DNAM-1, NKp30, NKp46, Perforin and Granzyme B were significantly lower after exposure SW1990 cells compared to NK cells cultured alone or co-cultured with normal pancreatic hTERT-HPNE cells. Similarly, the percentages of NK cells positive for DNAM-1, Perforin and Granzyme B were also significantly downregulated following exposure to BxPc-3 cells. However, there was no significant change in the percentages of NK cells positive for activating surface receptors (NKp44 and NKp80) or inhibitory surface receptors (KIR3DL1 and KIR2DL1/DS1) with either of the pancreatic cancer cell lines. The significances of these results are given in Figure 1 (*P* <0.05, *P* <0.01, or *P* <0.001). These data suggested that pancreatic cancer cells might induce NK cell dysfunction by targeting specific surface receptors and cytotoxic granules.

### Pancreatic cancer cells induced apoptosis and downregulated cytotoxic activity in NK cells

**Figure 2:**
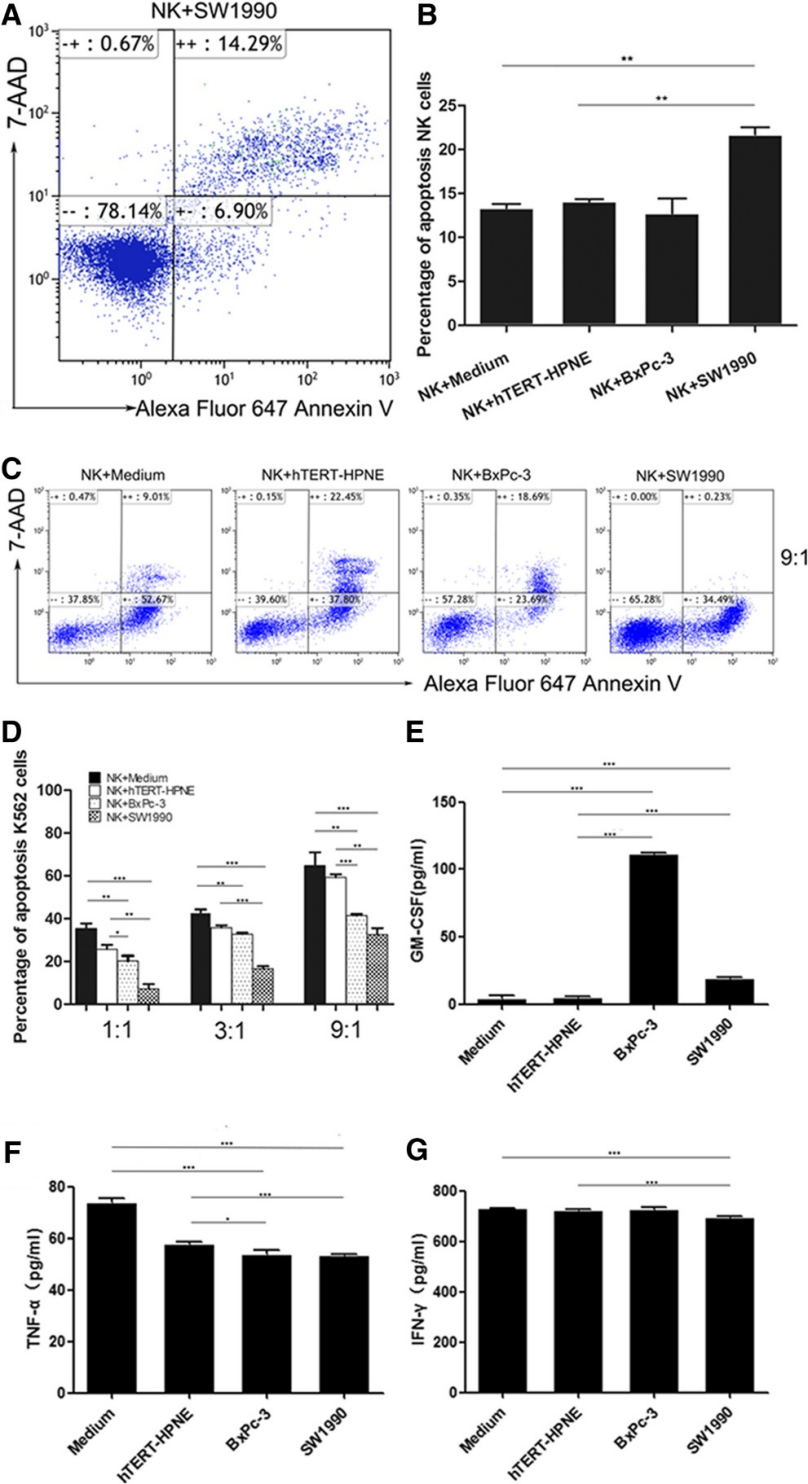
**The influence of pancreatic cancer cells on the apoptosis, cytotoxic activity, and cytokines secretion of NK cells.** **(A and** **B)** Percentages of apoptotic NK cells exposed to normal pancreatic cells compared those exposed to pancreatic cancer cells. **(C)** Flow cytometric analysis of the percentages of apoptotic K562 cells treated with NK cells, either cultured alone or with normal pancreatic cells or cancer cells, at an effector-to-target (E/T) ratio of 9:1. **(D)** The percentages of apoptotic K562 cells at different E/T ratios (1:1, 3:1, and 9:1) show a significant decrease in K562 cell apoptosis after treatment with NK cells exposed to pancreatic cancer cells compared to those exposed to normal pancreatic cells. **(E,** **F, and** **G)**. The concentrations of GM-CSF, TNF-α and IFN-γ in the supernatants from the different NK cell-pancreatic cell culture groups. All experiments were repeated three times. All data were expressed as mean ± SD. Independent t-tests were used for statistical analysis through SPSS 19.0 software. *represents P <0.05, **represents P <0.01, and ***represents P <0.001.

To investigate whether pancreatic cancer cells could induce apoptosis in NK cells, NK cells were analyzed after being cultured alone or co-cultured with normal pancreatic hTERT-HPNE cells or pancreatic cancer SW1990 or BxPc-3 cells. As shown in Figure 2A and B, the percentages of apoptotic NK cells following exposure to SW1990 cells was significantly higher compared to those cultured alone or with normal pancreatic hTERT-HPNE cells. However, no significant difference was observed following exposure to BxPc-3 cells.

The decreased percentages of NK cells positive for Perforin and Granzyme-B after exposure to SW1990 and BxPc-3 suggested that pancreatic cancer cells could reduce the cytotoxic activity of NK cells *in vitro*. Therefore, we investigated the cytotoxicity of NK cells against myelogenous leukemia K562 cells. As shown in Figure 2C and D, the cytotoxic activity of NK cells against K562 cells was significantly downregulated following exposure to SW1990 cells or BxPc-3 cells at different E/T ratios, compared to those cultured alone or with hTERT-HPNE cells. However, the negative effect of SW1990 cells was greater than that of BxPc-3 cells. These findings suggested that pancreatic cancer cells could negatively influence the cytotoxic activity of NK cells. This was most apparent with SW1990 cells.

### Pancreatic cancer cells altered the concentration of cytokines secreted by NK cells

In order to further understand the functional status of NK cells following exposure to pancreatic cancer cells, the concentrations of cytokines (GM-CSF, TNF-α and IFN-γ) in the supernatants following cell culture were determined by ELISA. As shown in Figure 2E to G, the concentrations of GM-CSF secreted by NK cells following exposure to BxPc-3 and SW1990 cells was significantly increased, as determined by the lower levels of TNF-α, compared to NK cells cultured alone or with normal pancreatic hTERT-HPNE cells; however, the expression of IFN-γ was only found to be downregulated in NK cells that were exposed to SW1990 cells. These data suggest that pancreatic cancer cells might also induce NK cell dysfunction by impairing the secretion of cytokines.

### Overexpression of MMP-9 and IDO in SW1990 cells treated with NK cells

**Figure 3:**
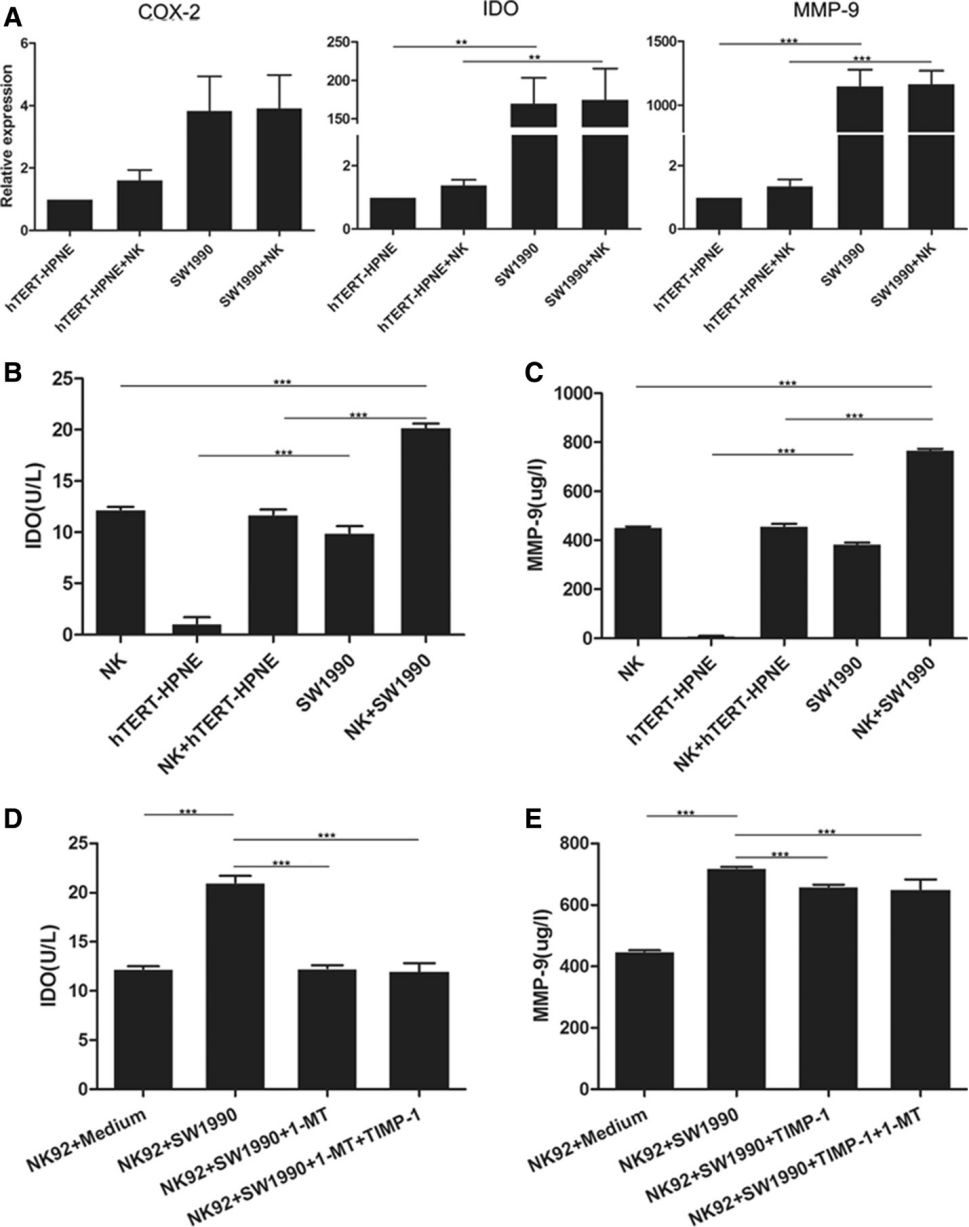
**The expression of COX-2, MMP-9 and IDO in pancreatic cancer cells.** **(A)** Relative expressions of COX-2, MMP-9 and IDO in hTERT-HPNE cells compared to SW1990 cells cultured alone or co-cultured with NK cells. **(B and** **C)** The concentrations of IDO and MMP-9 in cell cultural supernatants from NK cells cultured alone, hTERT-HPNE cells cultured alone or co-cultured with NK cells, SW1990 cells cultured aloneor co-cultured with NK cells. **(D and** **E)** The corresponding plots for NK-92 cells cultured alone or cultured with SW1990, in the absence or presence of 0.5 mM 1-MT and/or 0.5 ug/ml TIMP-1, show that the concentrations of IDO and MMP-9 can be partially restored by these blockers.All experiments were repeated three times. All data were expressed as mean ± SD. Independent t-tests were used for statistical analysis through SPSS 19.0 software. *represents P <0.05, **represents P <0.01, and ***represents P <0.001.

Although we had demonstrated that pancreatic cancer cells could induce NK cell dysfunction, the mechanisms were unclear. Previous evidence had indicated that MMP-9, IDO and COX-2 might be implicated in the dysfunction of NK cells [16, 20], therefore the expressions of these proteins in SW1990 cells and hTERT-HPNE cells cultured alone or co-cultured with NK cells was investigated. Real-time qRT-PCR showed that the relative expression of MMP-9 and IDO was significantly higher in SW1990 cells compared to normal pancreatic hTERT-HPNE cells. However no significant difference was observed in the expression levels of COX-2 (Figure 3A). ELISA also showed that the concentrations of MMP-9 and IDO was markedly higher in supernatants from SW1990 cells cultured alone compared to hTERT-HPNE cells cultured alone; the concentrations of MMP-9 and IDO was also higher in supernatants from SW1990 cells co-cultured with NK cells compared to hTERT-HPNE cells co-cultured with NK cells or NK cells cultured alone (Figure 3B and C). These data suggested that MMP-9 and IDO might play a role in SW1990 cell-induced dysfunction of NK cells.

### Blocking MMP-9 and/or IDO restored SW1990 cell-induced NK-92 cell dysfunction

To further confirm that MMP-9 and IDO were implicated in SW1990 cell-induced NK cell dysfunction, NK-92 cells were exposed to SW1990 cells and treated with specific blockers for MMP-9 and IDO, TIMP-1 and 1-MT, respectively. NK-92 cells were selected as they are more stable and homogeneous than purified NK cells. ELISA showed that the concentrations of MMP-9 and IDO were increased in the supernatants from NK-92 cells co-cultured with SW1990 cells compared to NK-92 cells cultured alone, but decreased in the presence of TIMP-1 and/or 1-MT, respectively (Figure 3D and E).

**Figure 4:**
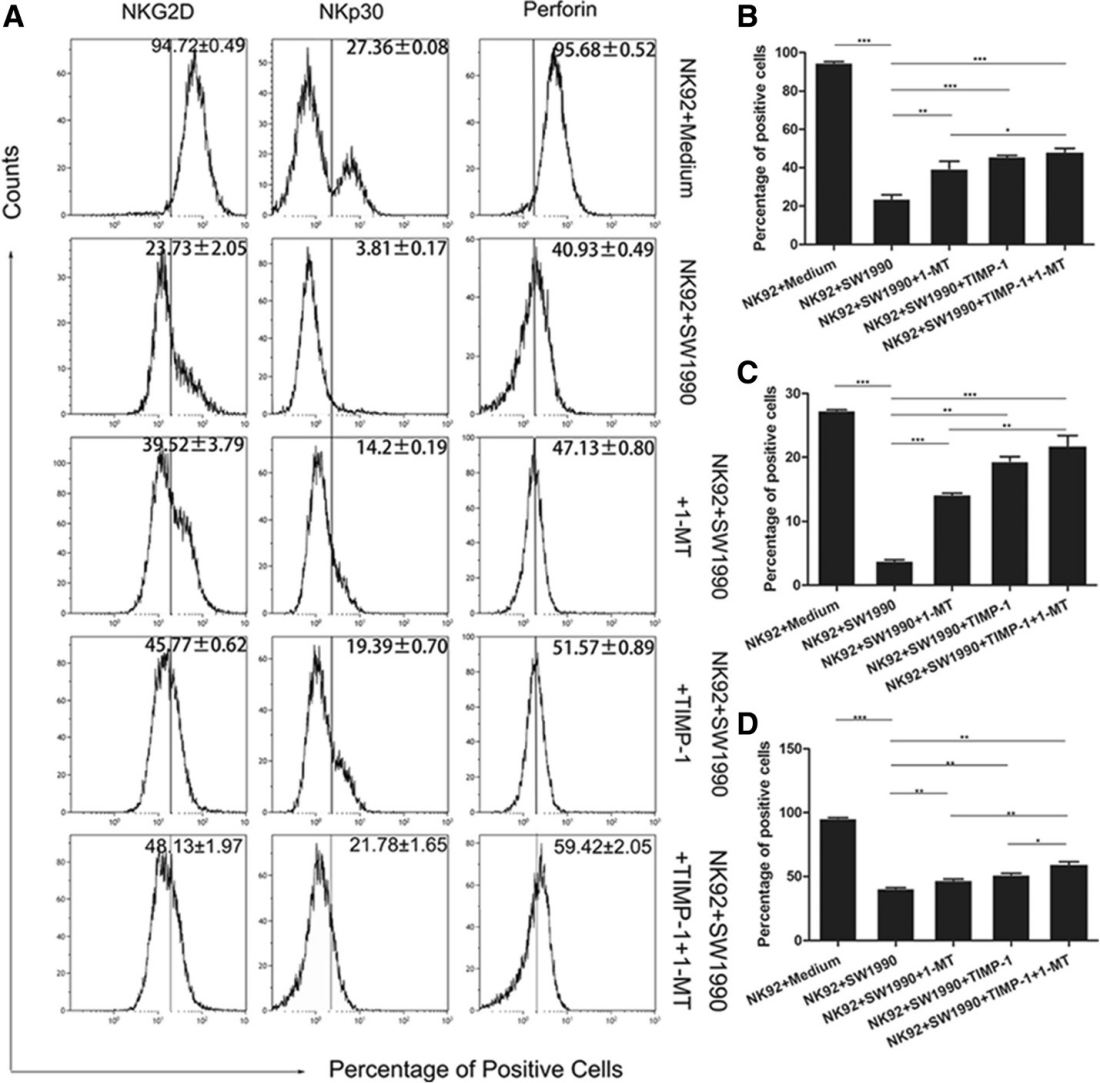
**The effect of MMP-9 and IDO blockers in NK-92 cells cultured alone or with SW1990 cells in the absence or presence of 1-MT and/or TIMP-1. (A)** The percentages of NKG2D-, NKp30- and Perforin-positive NK-92 cells in the different NK-92 cell-pancreatic cell treatment groups. **(B,** **C and** **D)** Comparisons between these different treatment groups demonstrate that the addition of 1-MT and/or TIMP-1 blockers can partially restore the percentage of positive NK-92 cells. All experiments were repeated three times. All data were expressed as mean ± SD. Independent t-tests were used for statistical analysis through SPSS 19.0 software. *represents P <0.05, **represents P <0.01, and ***represents P <0.001.

**Figure 5:**
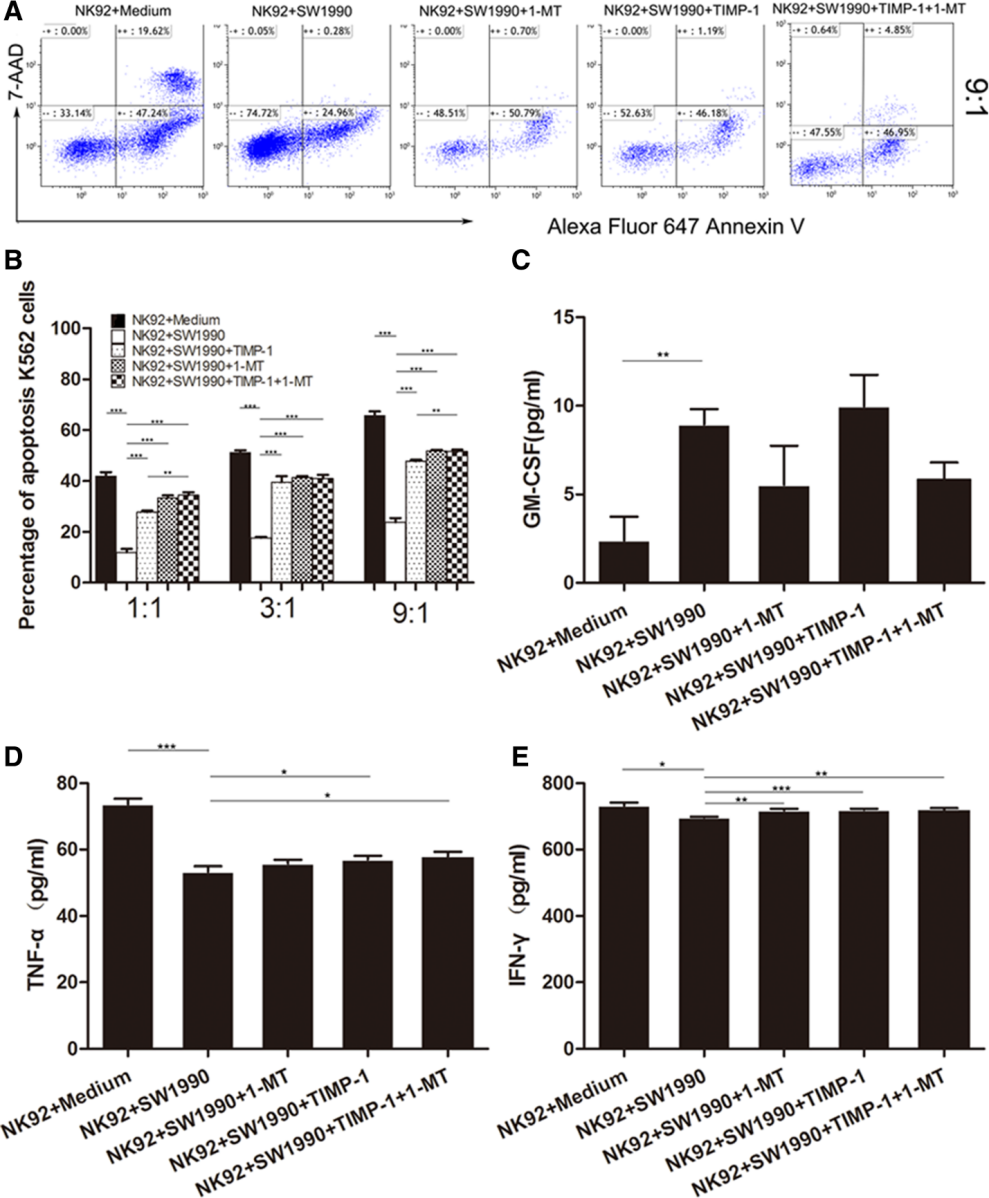
**Apoptosis in myelogenous leukemia K562 cells treated with NK-92 cells either cultured alone or with SW1990 cells in the absence or presence of MMP-9 and/or IDO blockers. (A)** Flow cytometric analysis of apoptosis in K562 cells treated with NK-92 cells with 1-MT and/or TIMP-1, at an effector-to-target (E/T) ratio 9:1. **(B)** The percentages of apoptotic K562 cells treated with NK-92 cells at different E/T ratios (1:1, 3:1 and 9:1), show that the addition of 1-MT and/or TIMP-1 blockers upregulates apoptosis in K562 cells. **(C,** **D, and** **E)** Comparisons between the concentrations of GM-CSF, TNF-α and IFN-γ in the supernatants from the different NK cell-pancreatic cell culture groups. All experiments were repeated three times. All data were expressed as mean ± SD. Independent t-tests were used for statistical analysis through SPSS 19.0 software. *represents P <0.05, **represents P <0.01, and ***represents P <0.001.

Furthermore, the percentages NK-92 cells positive for NKG2D, NKp30, NKp44, NKp46, DNAM-1, Perforin and Granzyme B following exposure to SW1990 cells was significantly downregulated compared to NK-92 cells cultured alone (Table 1). However, the levels of NKG2D-, NKp30- and Perforin-positive NK-92 cells could be partially restored following the introduction of TIMP-1 and/or 1-MT (Figure 4). A similar effect was observed when the cytotoxic activity of NK-92 cells was investigated. As shown in Figure 5A and B, blocking MMP-9 and/or IDO largely restored the cytotoxicity of NK-92 cells. The data also showed that blockade of MMP-9 by TIMP-1 could partially restore the secretion of TNF-α and IFN-γ (Figure 5D and E), and blockade of IDO by 1-MT could restore the secretion of TNF-α (Figure 5D). However, blockade by either MMP-9 and/or IDO showed any significant effect on the level of GM-CSF. Furthermore, the effect of blocking MMP-9 and IDO simultaneously was significantly obvious in some aspects than that of blocking IDO individually, but was similar to that of blocking MMP-9 individually. Taken together, these data provided further evidence that MMP-9 and IDO may play key roles in SW1990 cell-induced NK-92 cell dysfunction, however, there are no obvious synergic effects of MMP-9 and IDO.

**Table 1:** **The percentages of surface receptor**- **and cytotoxic granule**-**positive NK**-**92 cells co**-**cultured with SW1990 compared to those cultured alone**

	NK-92 + Medium (%)	NK92 + SW1990 (%)	***P*** -value
**NKG2D**	94.72 ± 0.49	23.73 ± 2.05	<0.001
**NKp30**	27.36 ± 0.08	3.81 ± 0.17	<0.001
**NKp44**	24.33 ± 2.07	0.93 ± 0.48	<0.01
**NKp46**	11.76 ± 1.14	1.73 ± 1.03	<0.01
**DNAM**-**1**	51.35 ± 2.06	2.63 ± 1.13	<0.001
**KIR3DL1**	2.06 ± 0.65	1.78 ± 1.19	Ns
**KIR2DL1**/**DS1**	1.79 ± 0.96	2.50 ± 0.85	Ns
**NKp80**	92.67 ± 2.74	91.61 ± 2.85	Ns
**Perforin**	95.68 ± 0.52	40.93 ± 0.49	<0.001
**Granzyme B**	95.81 ± 1.96	86.24 ± 3.01	<0.01

All experiments were repeated three times. All data were expressed as mean ± SD. Independent *t*-tests were used for statistical analysis through SPSS 19.0 software. *P* < 0.01 is considered significant; *P* < 0.001 is considered highly significant.

## Discussion

In a previous clinical trial, we found that the development of pancreatic cancer was closely correlated with tumor immune escape, mediated by dysfunction in the circulating NK cells [28]. In this study, we found that NK function was inhibited after exposure to pancreatic cancer cells *in vitro*. This effect was greatest with SW1990 cells. Further investigation showed that MMP-9 and IDO were overexpressed in SW1990 cells and acted as negative factors in SW1990 cell-induced NK cell dysfunction.

The association between NK cell dysfunction and altered expression of surface receptors, cytotoxic granules and cytokines has previously been reported in other cancers, including melanoma and cervical cancer [29, 30]. Activating surface receptors induce cell stress apoptosis by binding to specific ligands that are overexpressed on the surface of stressed cells, including cancer cells. For example, MHC class I polypeptide-related sequence A/B (MICA/MICB) and UL16-binding protein (ULBP) bind to NKG2D [31]; whereas cellular heparin or heparin sulfate proteoglycans bind to NCRs [32]. The cytotoxic granules Perforin and Granzyme-B cooperatively combine to form a complex which is released into the cytoplasm of infected cells and tumor cells [33]. Cytokines TNF-α, IFN-γ and GM-CSF are abundantly secreted by NK cells [34] and influence the recruitment and function of other hematopoietic cells (such as T cells) in the early stage of innate immune responses [35].

Our findings showed that most of the surface receptors, cytotoxic granules and cytokines discussed above were significantly altered in NK cells following exposure to pancreatic cancer cells. For example, lower expressions of NKG2D, NKp30 and NKp46; downregulation of Perforin and Granzyme-B; and decreased secretion of TNF-α and IFN-γ. These data demonstrated that pancreatic cancer cells suppress NK cells through a variety of mechanisms. However, increased secretion of GM-CSF in NK cells may counteract the inhibiting effect of pancreatic cancer cells on NK cells [36].

Inhibitory surface receptors remain hyporesponsive in healthy bodies but are upregulated when the body suffers infection or cancer to enable activated NK cells to perform anti-tumor or anti-infection functions [37]. Two inhibitory surface receptors, KIR3DL1 and KIR2DL1/DS1, were investigated in this study, however we found no significant alteration in their expression in NK cells that following exposure to pancreatic cancer cells.

Investigations into the mechanisms by which SW1990 cells might modulate NK cells revealed that SW1990 cells produced high levels of negative soluble mediators when co-cultured with NK cells, including MMP-9 and IDO. Subsequent inhibition of MMP-9 and IDO could partially restore NK cell function, suggested that these mediators promoted pancreatic tumor growth by suppressing NK cells.

MMP-9 promotes cancer progression through a variety of mechanisms. Previous studies have reported that MMP-9 decreased apoptosis in cancer cells [38]; reduced proliferation and metastasis in cancer cells derived from MMP-9 deficient mice compared to those from wild-type mice [39, 40]; promoted angiogenesis in two transgenic models of cancer progression [38, 39]; inhibited T cell activity against tumors by enhancing IL-2Rα and TGF-β production [41]; and could significantly suppress the cytotoxicity of NK cells in oral squamous cell carcinoma cell lines [20]. In support of these studies, we showed that MMP-9 negatively influenced NK cell function through decreased expression of NKG2D, NKp30 and Perforin and inhibition of IFN-γ and TNF-α. Conversely, we showed that inhibition of MMP-9 largely restored the levels of NKG2D-, NKp30- and Perforin-positive NK-92 cells, the secretion of TNF-α and IFN-γ and the cytotoxicity on NK cells against myelogenous leukemia K562 cells. However, our investigation was unable to determine whether MMP-9 targeted NK cells directly or acted *via* other pathways; for example, through promotion of IL-2Rα and TGF-β, which would in turn suppress NK cells; or *via* stimulation of cytokines, such as interleukin-8 (IL-8), connective-tissue activating peptide-III (CTAP-III), platelet factor-4 (PF4) and growth-related oncogene-α (GRO-α), which have previously been shown to alter infiltration and migration of leukocytes [42].

IDO catalyzes the first and rate-limiting step in the kynurenine pathway of tryptophan catabolism. Previous studies have suggested that IDO contributes to immunotolerance in patients with autoimmune diseases and chronic infections [43, 44], and that IDO expressed by hepatocellular carcinoma-associated fibroblasts could induce NK cell dysfunction *in vitro*[16]. Further studies have shown that IDO can facilitate cancer cells to escape immune surveillance by NK cells in cervical cancer [45] and by regulatory T cells in pancreatic and breast cancer [46, 47]. Our data has shown that IDO may promote the SW1990 cell-induced NK cell dysfunction by reducing the proportion of NKG2D-, NKp30- and Perforin-positive NK-92 cells; and by downregulating the secretion of IFN-γ and the cytotoxicity of NK cells against K562 cells. These findings were similar to our results with MMP-9, and there are no obvious synergic effects of MMP-9 and IDO. Therefore, their relationship in relation to NK cell dysfunction warrants further study.

The blocking effect of MMP-9 and IDO was incomplete suggesting that there may be additional factors involved in SW1990 cell-induced NK cell dysfunction. These could include PGE2, IL-8 and B7-H1 which have been reported to be overexpressed and act as immunosuppressants by reducing the cytotoxicity of T cells and NK cells in several solid cancers [48–50]. Increasing our understanding of the underlying immunosuppressive mechanisms and pathways could facilitate the development of immune-dependent therapies for patients with specific cancers.

## Conclusions

In conclusion, our results have provided evidence that pancreatic cancer cells induce NK cell dysfunction, however NK function could be partially restored by blocking MMP-9 and/or IDO, suggesting that MMP-9 and IDO facilitate pancreatic cancer cells to evade immunosurveillance. Although further study will be required to determine the details of the mechanisms involved and the interrelation between these molecules, our findings could contribute to NK cell-based immunotherapy in patients with pancreatic cancer.

## Abbreviations

NK cell: Natural killer cell
MMP-9: Matrix metallopeptidase 9
IDO: Indoleamine 2, 3-dioxygnase
MSC: Mesenchymal stem cells
NCR: Natural cytotoxicity receptors
1-MT: 1-Methyl-DL-tryptopan
TIMP-1: Tissue inhibitor of metalloproteinases 1
PBMC: Peripheral blood mononuclear cells
ELISA: Enzyme-linked immunosorbent assay
qRT-PCR: Quantitative real-time reverse-transcription polymerase chain reaction.
